# Phagocytized Neutrophil Fragments in the Bone Marrow: A Phenomenon Most Commonly Associated with Hodgkin Lymphoma

**DOI:** 10.1155/2014/363854

**Published:** 2014-03-18

**Authors:** Michael A. Arnold, Samir B. Kahwash

**Affiliations:** Department of Pathology and Laboratory Medicine, Nationwide Children's Hospital, 700 Children's Drive, Columbus, OH 43205, USA

## Abstract

Bone marrow macrophages containing other cells, or large pieces of other cells, represent a distinctive feature of diseases such as Hemophagocytic Lymphohistiocytosis (HLH) and Rosai-Dorfman disease. We describe a distinct variation of phagocytic histiocyte morphology, featuring histiocytes containing predominantly fragments of neutrophil nuclei. We retrospectively reviewed initial bone marrow samples for Hodgkin lymphoma, Burkitt lymphoma, Ewing sarcoma, or evaluation for nonneoplastic conditions, scoring the presence or absence of the above-described histiocytes. We find that these histiocytes, which we term “fragmentophages,” are associated with staging marrow sampling for malignancy, especially Hodgkin lymphoma (Hodgkin lymphoma: 28/34 or 82.4%, Ewing sarcoma: 11/26 or 42.3%, Burkitt lymphoma: 4/13 or 30.8%). These cells are significantly less common in marrow samples for nonneoplastic conditions (4/21 or 19.0%). Fragmentophages are significantly associated with malignancy, especially Hodgkin lymphoma, and their recognition has the potential to provide a clue to an underlying malignancy.

## 1. Introduction

The histologic patterns of Hemophagocytic Lymphohistiocytosis (HLH) and Rosai-Dorfman disease are distinctive, each characterized by macrophages containing large parts of other cells. HLH is characterized by increased macrophage uptake and destruction of red blood cells, red blood cell precursors, and platelets, resulting in potentially lethal peripheral cytopenias. Rosai-Dorfman disease features the proliferation of distinctive, large histiocytic cells which accumulate usually in the sinuses of lymph nodes and contain predominantly intact lymphocytes. Both of these diseases can involve the bone marrow. Thus, the identification of bone marrow macrophages containing other cells in their cytoplasm evokes consideration of these diagnoses [1, 2]. We have observed a morphologically distinct pattern of macrophages containing mainly neutrophil nuclear fragments in bone marrow samples of children in association with malignancy. This pattern is important to distinguish from HLH and Rosai-Dorfman disease and has the potential to be a first clue to an associated malignancy.

## 2. Methods and Materials

A retrospective review of initial bone marrow samples and patient chart material over a 5-year period was performed in accordance with an Institutional Review Board approved protocol. Staging marrow samples represented patients treated at our pediatric center for Hodgkin lymphoma, Ewing Sarcoma, and Burkitt lymphoma or were initial bone marrow biopsies for nonneoplastic conditions. Marrow samples which were involved by the associated malignancy were excluded. The identified marrow samples included 34 patients with Hodgkin lymphoma (age 3 to 19 years, 15 males and 19 females), 13 patients with Burkitt lymphoma (age 2 to 18 years, 10 males and 3 females), 26 patients with Ewing sarcoma (age 4 to 37 years, 19 males and 7 females), and 21 patients who underwent initial marrow sampling for a nonneoplastic condition (age 1 to 18 years, 15 males and 6 females). On review of bone marrow samples, the presence or absence of macrophages containing predominantly neutrophils or neutrophil fragments was scored. *P*-values were calculated using the chi squared test.

## 3. Results

### 3.1. Macrophages Containing Neutrophils Represent a Distinct Histologic Pattern

**Figure 1:**
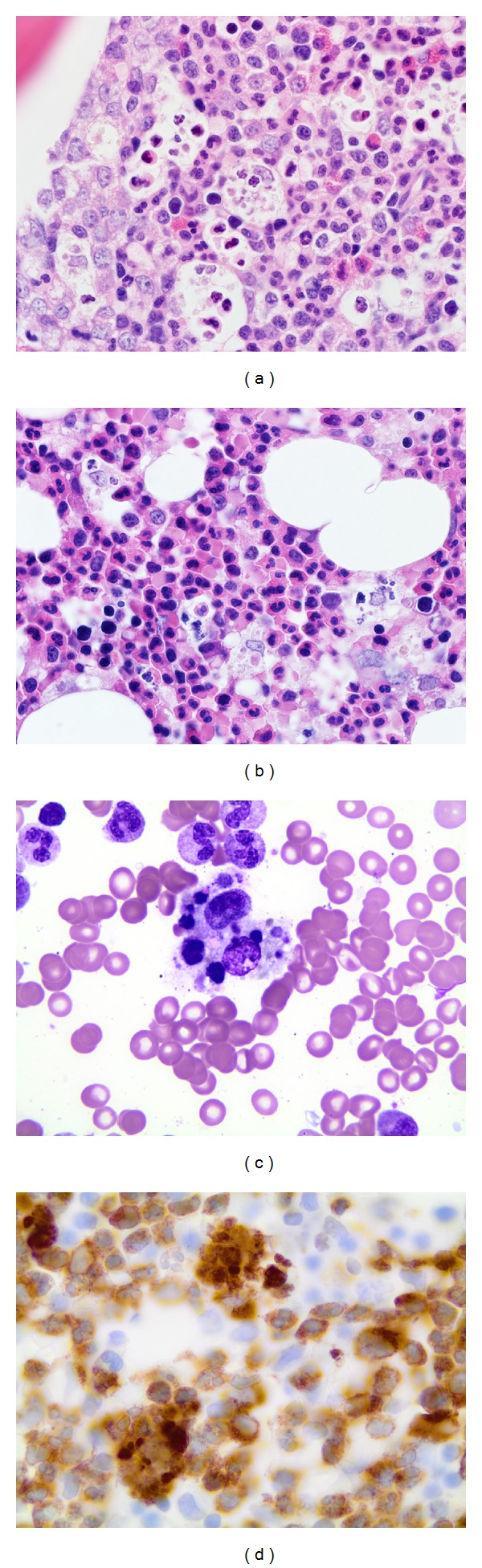
Macrophages containing granulocyte nuclear debris. Macrophages containing granulocyte nuclear debris can be seen dispersed throughout marrow biopsies in H&E stained sections ((a) H&E 40x objective magnification, (b) H&E 100x oil objective magnification) and aspirate marrow smears ((c) Wright-Giemsa 100x oil). Immunohistochemical staining for myeloperoxidase highlights the phagocytosed cells ((d) 100x oil). H&E: Hematoxylin and Eosin.

**Figure 2:**
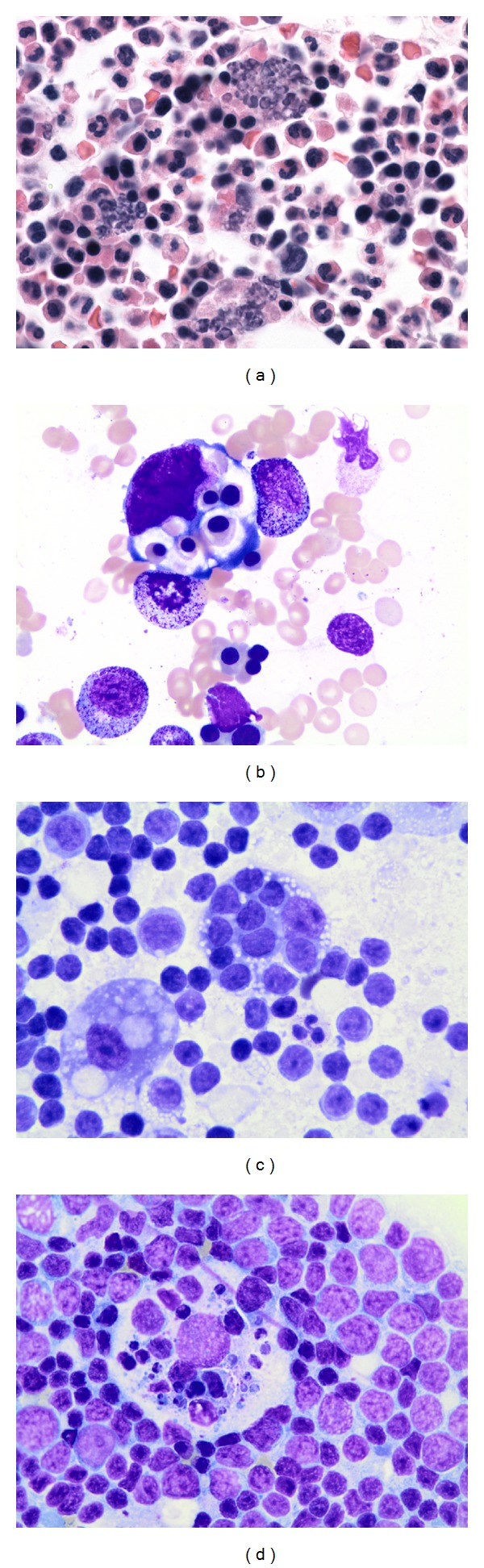
Distinct histologic features of macrophages containing other cell types. Macrophages containing neutrophils, or “fragmentophages,” show cytoplasmic inclusion of cells with lobulated nuclei ((a) H&E 100x oil). In contrast, HLH features macrophages containing red blood cells ((b) Wright-Giemsa 100x oil) and Rosai-Dorfman with macrophages containing intact lymphocytes ((c) Wright-Giemsa 100x oil). Tingible body macrophages are distinguished by cytoplasm which contains a range of fragments, from whole lymphocytes to small debris ((d) Wright-Giemsa 100x oil).

Macrophages containing neutrophils were observed in core biopsy sections (Figures 1(a) and 1(b)), as well as aspirate smears (Figure 1(c)). Immunohistochemical staining for myeloperoxidase highlights the cell fragments within these macrophages, further supporting that these cell fragments are derived from granulocytes (Figure 1(d)). The distinct morphology of these macrophages is easily distinguished on routine stains from other histologic types of macrophages. The macrophages we highlight all contain multiple granulocytes with lobulated nuclei, or fragments of similar appearing nuclei (Figure 2(a)). In contrast, HLH features macrophages containing predominantly erythropoietic cells with round nuclei, or their less distinct degradation products (Figure 2(b)). These patterns are also distinct from emperipolesis seen in Rosai-Dorfman disease, in which large histiocytic cells contain predominantly intact lymphocytes in their cytoplasms (Figure 2(c)). Each of these macrophage types is also readily distinguished from tingible body macrophages commonly encountered in reactive germinal centers (Figure 2(d)).

### 3.2. Macrophages Containing Neutrophils Are Associated with Malignancy

**Figure 3:**
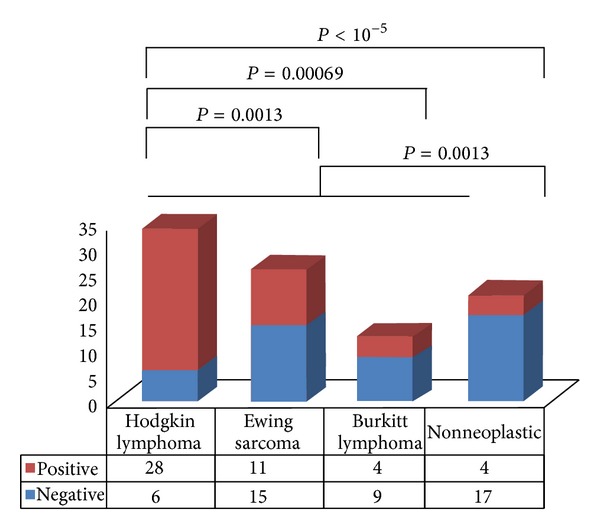
Distribution of bone marrow macrophages containing neutrophils by underlying diagnosis.

The characteristic histiocytes we observed were seen most often in staging marrow samples for Hodgkin lymphoma (82.4%) (Figure 3). These cells were also present in staging marrow samples for Ewing sarcoma (42.3%) or Burkitt lymphoma (30.8%) and were less frequent in bone marrow samples to evaluate nonneoplastic conditions (19.0%) (Figure 3). These characteristic macrophages are significantly associated with an underlying malignancy (odds ratio for any malignancy = 6.09, *P* = 0.0028; positive predictive value for malignancy = 91.5%, specificity for malignancy = 85%, sensitivity for malignancy = 58.9%), in particular with Hodgkin lymphoma (odds ratio for Hodgkin lymphoma versus nonneoplastic = 19.3, *P* < 0.0001; positive predictive value for Hodgkin lymphoma versus nonneoplastic = 87.5%, specificity for Hodgkin lymphoma = 81.0%, sensitivity for Hodgkin lymphoma = 82.4%). The association of these macrophages with Hodgkin lymphoma is highly significant compared to biopsies for nonneoplastic conditions (*P* < 10^−5^), and the other malignancies examined (Figure 3). Similarly, the less frequent occurrence of these cells in bone marrow biopsies for nonneoplastic conditions is significant when comparing staging bone marrow biopsies with the malignancies we examined (*P* = 0.0013) (Figure 3).

### 3.3. Macrophages Containing Neutrophils Occurred Independent of GCSF Administration

Since the characteristic macrophages we describe are seen to engulf neutrophils, and cytokines are known to be elevated in patients with Hodgkin lymphoma [3–6], we speculated that Granulocyte Colony Stimulating Factor (GCSF) might contribute to the formation of these cells. We examined the clinical charts of the 4 patients found to have these cells in their bone marrow biopsies for nonneoplastic indications. The indications for marrow biopsy in these patients were juvenile arthritis, thrombocytopenia, chronic neutropenia, and peripheral platelet destruction. We found no evidence of previous GCSF administration for any of these four patients. We conclude that the origin of these characteristic macrophages is independent of exogenous GCSF administration.

## 4. Discussion

We report a distinct pattern of macrophage morphology which is easily distinguished from the macrophages characteristic of HLH and Rosai-Dorfman disease, as well as tingible body macrophages. These macrophages engulf predominantly neutrophils or fragments of neutrophils, and we find that these cells are significantly associated with malignancy, in particular Hodgkin lymphoma. During the 5-year period we examined, we found that these macrophages were more than 6 times as likely to occur in the setting of the malignancies we examined when compared to bone marrow biopsies for nonneoplastic conditions. Additionally, these cells were more than 19 times more likely to be seen in initial bone marrow biopsies for Hodgkin lymphoma than in initial biopsies for nonneoplastic conditions.

Further systematic characterization of this intriguing morphologic finding is required to determine if this association is restricted to the malignancies we examined in children, or if these macrophages are universally associated with malignancy. Correlation with bone marrow biopsies in adults may be important, as it is possible that the inherently more brisk physiologic reactions in children contribute to the production of these macrophages. In this series, however, we did not identify a correlation between the occurrence of these macrophages and patient age.

Awareness of this morphologic pattern will be a key to further elucidate its potential clinical significance. We therefore propose utilizing a uniform nomenclature to facilitate the further description of these distinctive macrophages containing predominantly neutrophils, such as the term “fragmentophages.” While phagocytosis in the bone marrow has been vaguely mentioned in older medical literature on bone marrow findings in Hodgkin lymphoma [7], and as a part of “nonspecific histologic abnormalities of bone marrow” in Hodgkin lymphoma patients [8], the distinct histologic variant we highlight has not been specifically described. The use of nonspecific descriptive terms such as “phagocytosis of nuclear debris” may explain the lack of literature describing this specific morphologic pattern. Systematic and uniform reporting of this finding will facilitate its further description and will be necessary to determine if this distinct histologic pattern is a paraneoplastic phenomenon associated with other types of malignancy.

The mechanism which drives the formation of these cells is unclear. Hodgkin lymphoma is known to be associated with increased production of cytokines [3–6]. We suspect that the formation of these cells is driven by an inflammatory mediator associated with malignancy and that the engulfed neutrophils likely arise from myeloid hyperplasia in the bone marrow. We initially speculated that patients biopsied for nonneoplastic indications who were found to have these cells in their marrow biopsies may have previously received exogenous GCSF. However, on review of their chart material and medication administration records, GCSF was not mentioned for any of these 4 patients. These patients did not appear to follow a specific clinical pattern. We found these macrophages in bone marrow biopsies performed for juvenile arthritis, chronic neutropenia, thrombocytopenia, and peripheral platelet destruction. There is no clear inflammatory mediator in common among these conditions. It remains possible that an endogenous inflammatory mediator, which is particularly associated with Hodgkin lymphoma, is responsible for the generation of these characteristic bone marrow macrophages. We believe that awareness of this unique macrophage morphology, and its uniform description with a term such as “fragmentophages,” is key to distinguishing these cells from other types of macrophages, as well as better characterizing and understanding their association with malignancy.
